# Fidaxomicin for Initial Episode of *Clostridioides difficile* Infection Reduces Recurrence in Immunocompromised Hosts—a Large Retrospective Cohort Study

**DOI:** 10.1093/ofid/ofaf751

**Published:** 2025-12-11

**Authors:** Alaa Atamna, Adi Turjeman, Genady Drozdinsky, Haim Ben Zvi, Tzippy Shochat, Jihad Bishara

**Affiliations:** Infectious Diseases Unit, Rabin Medical Center, Beilinson Hospital, Petah-Tikva, Israel; Faculty of Medical and Health Sciences, Tel-Aviv University, Tel-Aviv, Israel; Faculty of Medical and Health Sciences, Tel-Aviv University, Tel-Aviv, Israel; Rabin Medical Center, Beilinson Hospital, Petah-Tikva, Israel; Infectious Diseases Unit, Rabin Medical Center, Beilinson Hospital, Petah-Tikva, Israel; Faculty of Medical and Health Sciences, Tel-Aviv University, Tel-Aviv, Israel; Faculty of Medical and Health Sciences, Tel-Aviv University, Tel-Aviv, Israel; Clinical Microbiology Laboratory, Rabin Medical Center, Beilinson Hospital, Petah-Tikva, Israel; Faculty of Medical and Health Sciences, Tel-Aviv University, Tel-Aviv, Israel; Beilinson Hospital, Rabin Medical Center, Petah-Tikva, Israel; Infectious Diseases Unit, Rabin Medical Center, Beilinson Hospital, Petah-Tikva, Israel; Faculty of Medical and Health Sciences, Tel-Aviv University, Tel-Aviv, Israel

**Keywords:** *Clostridioides difficile*, immunocompromised, recurrence

## Abstract

**Introduction:**

Fidaxomicin is a narrow-spectrum, nonabsorbable antibiotic recommended as first-line therapy for nonfulminant *Clostridioides difficile* infection (CDI). While some studies suggest a reduction of recurrence for immunocompromised hosts (ICHs), the findings remain inconsistent and limited. We aimed to evaluate the clinical outcomes of fidaxomicin for a first CDI episode in ICHs within a large healthcare system.

**Methods:**

We conducted a retrospective cohort study of Clalit Health Services adults with a first laboratory-confirmed CDI between January 2013 and December 2023. Patients with prior CDI were excluded. The primary outcome was a composite of recurrence, 90-day mortality, and colectomy, which were compared between the fidaxomicin and vancomycin groups. Secondary outcomes included each component individually.

**Results:**

The study included 11 204 patients with a first CDI episode, of whom 2362 were immunocompromised. The fidaxomicin group had a significantly lower recurrence rate compared to vancomycin (8% vs 17%, *P* = .019), while it was not associated with reduced composite outcome (aOR = .65, 95% CI .39–1.07, *P* = .09). Multivariable analysis also confirmed a reduction in recurrence risk (aOR 0.44, 95% CI .21–.96). Older age, previous antibiotic use within 90 days, and hypoalbuminemia were independently associated with worse outcomes, while recurrence was linked to increased long-term mortality.

**Conclusions:**

ICHs treated with fidaxomicin had significantly lower recurrence rates and fewer complications compared to vancomycin. Despite assumptions, ICHs had recurrence rates similar to immunocompetent patients, highlighting the role of comorbidities and treatment choices. Our findings support fidaxomicin as a preferred first-line option in select ICHs and underscore the need for individualized management strategies to improve outcomes in this high-risk population.

Fidaxomicin is a novel, nonabsorbable, bactericidal macrocyclic antibiotic used in the treatment of *Clostridioides difficile* infection (CDI). It has a narrower spectrum of activity compared to vancomycin, resulting in less disruption of the gut microbiota and, consequently, a lower rate of CDI recurrence [1].

In a focused update released in June 2021, the Infectious Diseases Society of America (IDSA) and the Society for Healthcare Epidemiology of America (SHEA) recommended fidaxomicin as a first-line therapy for nonfulminant CDI [2]. However, its higher cost and limited insurance coverage continue to pose barriers to widespread patient access.

Immunocompromised hosts (ICHs) experience a significantly higher incidence and recurrence rate of CDI compared to the general population [3]. This is primarily due to frequent antimicrobial exposure, immunosuppression, and increased contact with healthcare environments [3].

The CDI recurrence rate in this population show wide variability, reaching up to 40% in patients with hematologic malignancies and ranging from 8% to 41% among hematopoietic stem cell transplant (HSCT) recipients [3, 4]. In solid organ transplant (SOT) recipients, recurrence rates range from 12% to 40%, depending on the type of organ transplanted [5–7].

A recent multicenter study conducted in Spain, Italy, and Israel reported an overall CDI recurrence rate of 12% in SOT, with lung transplant recipients exhibiting the highest rate at 18% [8].

Currently, there are no specific guidelines for the treatment of CDI in ICHs, likely due to limited data in this population. Only a few studies have investigated the efficacy of fidaxomicin in ICHs with conflicting results. A post hoc analysis of patients with cancer and CDI found a significantly lower recurrence rate in those treated with fidaxomicin compared to vancomycin (13.5% vs 29.6%; *P* = .02) [9]. Another retrospective study of ICHs with CDI, found that fidaxomicin significantly reduced the combined risk of 30 and 90-day relapse (hazard ratio 0.27, 95% CI: .08–.91) [10]. In contrast, a retrospective study that included HSCT and SOT recipients found that the recurrence rates did not differ significantly between those treated with fidaxomicin and those receiving conventional therapy (7%, vs 7%; *P* = 1) [11].

Given the limited and conflicting evidence, we sought to evaluate whether ICHs with a first episode of CDI had improved clinical outcomes when treated with fidaxomicin, within the largest healthcare provider in Israel.

## MATERIALS AND METHODS

### Data Source

This study utilized data from the Clalit Health Services (CHS) database. CHS is the largest healthcare provider in Israel, providing comprehensive healthcare to over half of Israel's population (∼4.7 million individuals). In Israel, healthcare coverage is universal and mandatory under the National Health Insurance Law (1995).

The CHS database integrates information from various sources, including primary care physicians, community specialty clinics, hospital records, laboratories, and pharmacies. It supports a registry of chronic disease diagnoses compiled from these inputs. Diagnoses are identified using condition-specific algorithms based on ICD-10 codes, laboratory results, and the use of disease-specific medications. For each diagnosis, the database logs the sources and dates of the contributing data, designating the earliest date from any source as the official date of diagnosis. Data for this study were obtained from Clalit Health Services using the data-sharing platform powered by MDClone (https://www.mdclone.com).

### Study Population

The study cohort consisted of all CHS adult members aged 18 years or older with a first laboratory confirmed CDI diagnosis (index CDI episode) between January 2013 and December 2023. We excluded patients with a prior diagnosis of CDI. In addition, we excluded patients who required an extended initial course or a switch in regimen due to lack of improvement.

### Definitions

#### First CDI Episode

Date of first positive stool assay for *C. difficle* that was associated with documentation of CDI antibiotics initiation. The stool assay was considered positive when both *C. difficile* glutamate dehydrogenase (GDH) and toxin A/B ELISA assay were positive, or PCR for *C. difficile* toxin gene positive in case of *C. difficle* GDH antigen was positive and toxin A/B antigen negative [12]. CDI treatment patients were classified as being treated with either fidaxomicin, vancomycin, or metronidazole if they received at least 72 h of one of these agents.

#### CDI Recurrence

Re-initiation of CDI antibiotics within 8 weeks after treatment completion of the first CDI episode.

#### Immunocompromised Host

Organ transplant recipients (solid organ or HSCT), active solid tumor, active hematologic malignancy, human immunodeficiency virus/acquired immunodeficiency syndrome (HIV/AIDS), other immunocompromising condition treated with immunosuppressive drugs (Supplementary Table 1 and Table 2, see Appendix).

### Outcomes

The primary outcome was a composite of CDI recurrence within 8 weeks, 90-day mortality, or colectomy within 8 weeks of the first CDI episode. Secondary outcomes included CDI recurrence within 8 weeks, colectomy within 8 weeks, and 90-day mortality.

### Statistical Analysis

Continuous variables are summarized with medians and interquartile range (IQR), and categorical variables are presented as numbers and proportions. We performed two main comparison analyses, first between immunocompetent and immunocompromised patients with CDI, and second, between two treatment groups (fidaxomicin vs vancomycin) in ICHs only using the chi-square test for categorical variables and using the Mann-Whitney-test for continuous variables. Univariable analysis of risk factors for the primary composite outcome was performed. Multivariable analysis was adjusted for age, Charlson score index, previous hospitalization within 90 days, previous abdominal surgery, previous exposure to antibiotics, and hypoalbuminemia.

The study was approved by the institutional review board of Rabin Medical Centre and Data Utilization Committee of Clalit Health Services (CHS). Owing to the retrospective nature of the study, a waiver of informed consent was granted by the Institutional Review Board. The current study followed the Strengthening Reporting of Observational Studies in Epidemiology (STROBE) reporting guideline.

## RESULTS

**Figure 1.**
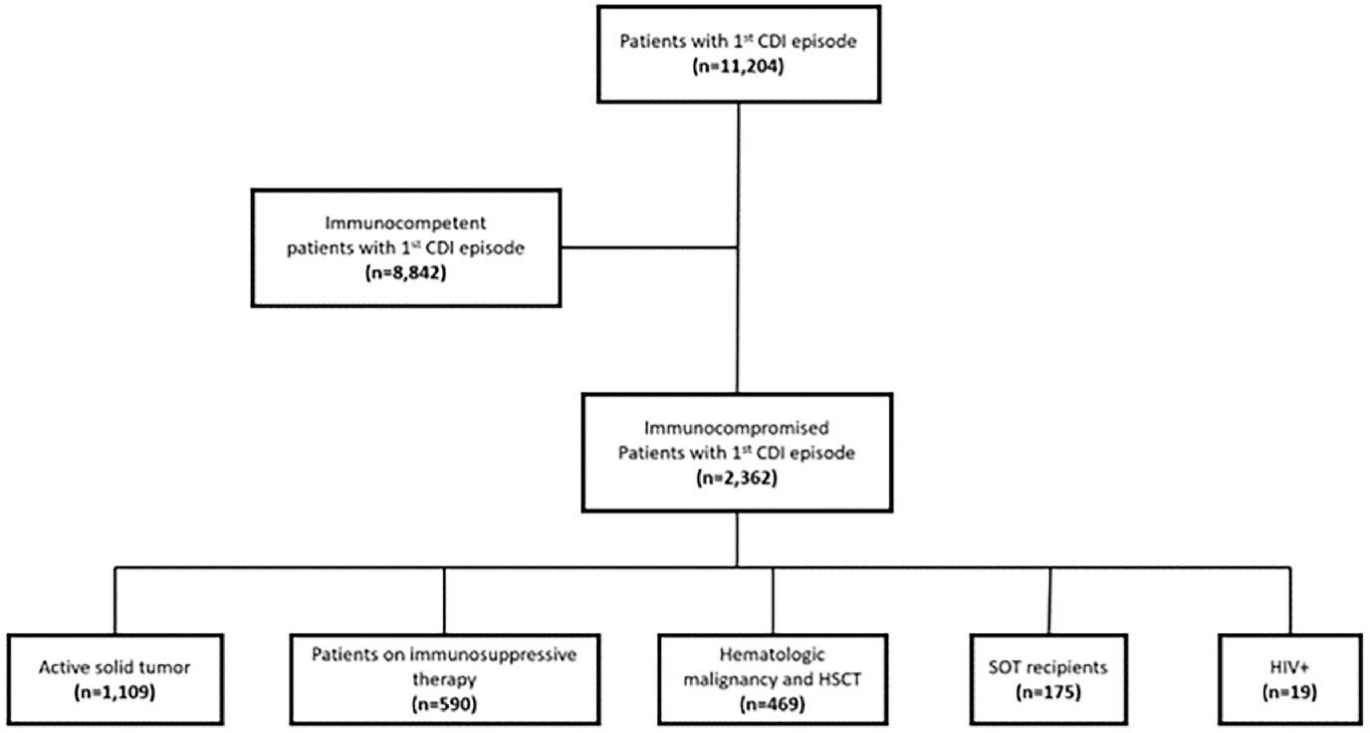
Study flowchart.

The study included 11 204 patients with a first episode of CDI, of which 2362 were ICHs. Figure 1 shows the flow chart of the study. The ICH group consisted of patients with active solid tumors 47% (1109/2362), patients with hematologic malignancies and HSCT recipients 20% (469/2362), solid organ transplant (SOT) recipients 7% (175/2362), and patients with immunocompromising conditions using immunosuppressive medications 25% (590/2362), such as immunomodulators, small molecule drugs, monoclonal antibodies, and long-term use of high dose corticosteroids (Supplementary Figure 1 and Supplementary Table 2).

### *Clostridioides difficle* Infection in Immunocompromised Hosts

Baseline characteristics of patients with a first CDI episode among ICHs are summarized in Table 1. Compared to immunocompetent patients, ICHs were younger but had a higher burden of comorbidities (median age of 68 vs 74 years, *P* < .01; median Charlson comorbidity index of 7 vs 6, *P* < .01). A higher proportion of ICHs were diagnosed with CDI in the hospital setting compared to immunocompetent patients 56% (1325/2362) versus 37% (3248/8842), *P* < .01. Known risk factors for CDI were more frequently observed among ICHs than immunocompetent patients, including prior hospitalization within 90 days of CDI diagnosis 23% (553/2362) versus 21% (1882/8842), *P* = .03, previous antibiotic exposure within 90 days 67% (1572/2362) versus 60% (5302/8,842, *P* < .001), and treatment with proton pump inhibitors 60% (1426/2362) versus 44% (3920/8842), *P* < .001.

**Table 1.** Baseline Characteristics of Patients With CDI According to Immunity Status

Variables	Immunocompromised (n = 2362)	Immunocompetent (n = 8842)	*P* Value
Age, median (IQR)	68 (56–78)	74 (56–84)	<.01
Age ≥ 65 y, n (%)	1378 (58%)	5758 (65%)	<.01
CCI, median (IQR)	7(4–10)	6 (2–9)	<.01
CCI ≥ 3 points, n (%)	1974 (84%)	6601 (75%)	<.01
Male gender, n (%)	1353 (57%)	5551 (63%)	<.01
**Referring facility**	**…**	**…**	
Hospital, n (%)	1325 (56%)	3248 (37%)	<.01
Community, n (%)	963 (41%)	4879 (55%)	
Nursing home, n (%)	74 (3%)	715 (8%)	
**Ethnicity**	…	…	
Jews, n (%)	2141 (91%)	8139 (92%)	.03
Arab, n (%)	221 (9%)	703 (8%)	
**Socioeconomic**	…	…	
High, n (%)	593 (25%)	1928 (22%)	.002
Low, n (%)	291 (12%)	1201 (14%)	
**CDI test method**	…	…	
Immunoassay, n (%)	1347 (57%)	6157 (70%)	<.001
PCR, n (%)	1012 (43%)	2681 (30%)	
Nap1, n (%)	3 (0.1%)	4 (0.05%)	
Hypertension, n (%)	1473 (62%)	5766 (65%)	.01
Diabetes mellitus, n (%)	926 (39%)	3403 (38%)	.53
Ischemic heart disease, n (%)	697 (30%)	2850 (32%)	.01
Chronic kidney disease, n (%)	851 (36%)	2681 (30%)	<.001
Hemodialysis, n (%)	228 (9.6%)	431 (5%)	<.001
Pre-hospitalization within 90 d, n (%)	553 (23%)	1882 (21%)	.03
Prior abdominal surgery, n (%)	70 (3%)	116 (1.3%)	<.001
Previous antibiotic exposure within 90 d, n (%)	1572 (67%)	5302 (60%)	<.001
PPI treatment, n (%)	1426 (60%)	3920 (44%)	<.001

Abbreviations: IQR, interquartile range; CCI, Charlson comorbidity index; CDI, *C. difficle* infection; PCR, polymerase chain reaction; NAP1, north American pulsed field gel electrophoresis type 1; PPI, proton pump inhibitor.

**Table 2.** CDI Clinical Course and Outcomes

Variables	Immunocompromised (n = 2362)	Immunocompetent (n = 8842)	*P* Value
Concurrent non-CDI antibiotics, n (%)	880 (37%)	2435 (28%)	<.001
WBC count (K/µL), median (IQR)	9 (5.8–13.3)	10.4(7.6–15.2)	<.01
WBC count ≥15 K/µL, n (%)	320 (13.5%)	1261 (14.3%)	<.01
Creatinine level (mg/dL), median (IQR)	.95 (0.70–1.53)	0.91 (0.68–1.50)	0.2
Albumin level (mg/dL), median (IQR)	2.93(2.5–3.45)	2.90(2.5–3.5)	.4
Albumin level ≤3 mg/dL, n (%)	720 (30%)	2011 (23%)	.9
CRP level (mg/dL), median (IQR)	8.8 (3–18)	9 (2.3–19)	.8
Fidaxomicin treatment, n (%)	107 (5%)	47 (0.5%)	.01
Vancomycin treatment, n (%)	994 (42%)	2820 (32%)	<.001
Metronidazole treatment, n (%)	677 (29%)	2702 (31%)	<.001
Recurrent CDI, n (%)	347 (15%)	1423 (16%)	.08
Post CDI colectomy, n (%)	20 (0.8%)	49 (0.5%)	.13
30-d mortality, n (%)	126 (5%)	485 (5%)	.8
90-d mortality, n (%)	379 (16%)	1322 (15%)	.2

Abbreviations: CDI, *C. difficle* infection; WBC, white blood cell; SD, standard deviation; CRP, C reactive protein; IQR, interquartile range.

At the time of CDI diagnosis, there were no significant differences in laboratory parameters between ICHs and immunocompetent patients with regard to creatinine (1.46 ± 1.54 vs 1.48 ± 1.65, *P* = .2), albumin (median 2.93 vs 2.90, *P* = .4), or C reactive protein (CRP) (median 8.8 vs 9, *P* = .8). However, immunocompetent patients had significantly higher white blood cell counts (median 10.4 K vs 9 K, *P* < .01).

In terms of treatment, the majority of ICHs with a first CDI episode received vancomycin 42% (994/2362), followed by metronidazole 29% (677/2362). Only 5% (107/2362) received fidaxomicin.

There was no significant difference in CDI recurrence rates between ICHs and immunocompetent patients 15% (347/2362) versus 16% (1432/8842), and the median time to first CDI recurrence was similar between the two groups 28 days (21–39 IQR) versus 27 days (21–37 IQR). Baseline characteristics, clinical course, and outcomes for the specific immunocompromising condition categories are summarized in Supplementary Table 3.

**Figure 2.**
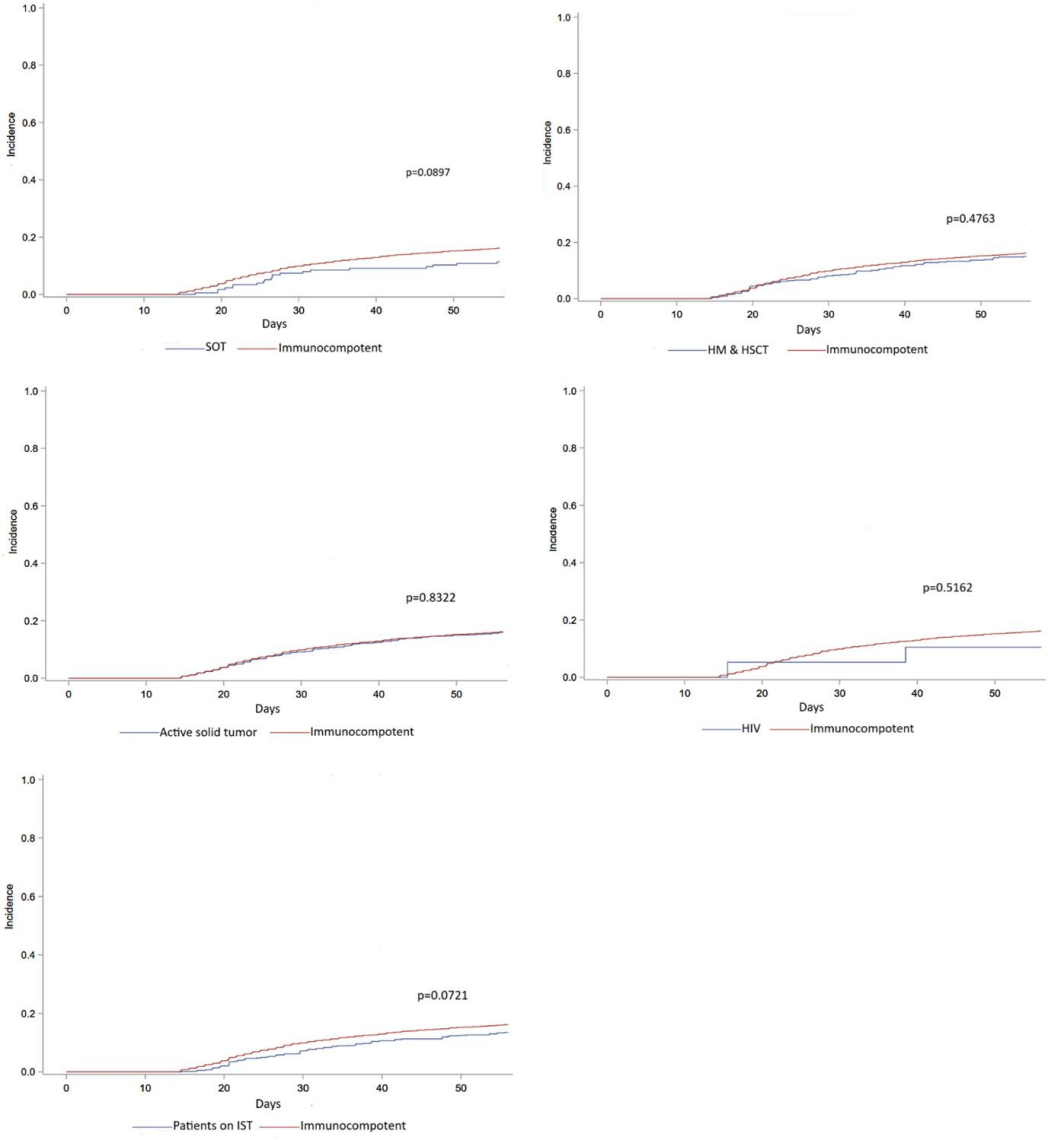
Incidence of CDI recurrence within 8 wks after 1st CDI episode in immunocompetent and immunocompromised hosts.

Figure 2 *A* shows the cumulative incidence of CDI recurrence within 8 weeks following the first CDI episode among immunocompetent and ICHs. Figure 2*B* through F display the cumulative incidence of CDI recurrence within 8 weeks by each immunocompromised condition category.

### Fidaxomicin versus Vancomycin for First Episode *C. difficle* Infection in Immunocompromised Hosts

A total of 107 ICHs were treated with fidaxomicin for their first CDI episode. Patients in this group were younger than those treated with vancomycin (median age 64 vs 68 years, *P* = .02) and were more likely to have higher socioeconomic status 34% (37/107) versus 25% (252/994), *P* = .03. They were also more likely to have had previous antibiotic exposure within 90 days 83% (89/107) versus 70% (693/994), *P* = .003, more likely to be diagnosed in the hospital setting 79% (85/107) versus 67% (665/994), *P* = .009, and more frequently diagnosed using toxin immunoassay 62% (66/107) versus 53% (522/994), *P* = .01.

Fidaxomicin was more often prescribed to patients with active solid tumors 60% (64/107) versus 47% (464/994) and SOT recipients 12% (13/107) versus 8% (81/994), *P* = .02. Conversely, patients treated with vancomycin were more likely to have hematologic malignancies and be HSCT recipients 20% (202/994) versus 14% (15/107), *P* = .02 or have other immunocompromising conditions requiring immunosuppressive treatment 24% (240/994) versus 14% (15/107), *P* = .02. None of the patients with HIV received fidaxomicin (Table 3).

**Table 3.** Baseline Demographics and Clinical Characteristics of Immunocompromised Patients by Treatment Group

Variables	Fidaxomicin Group (n = 107)	Vancomycin Group (n = 994)	*P* Value
Age, median (IQR)	64 (53–72)	68 (55–78)	.02
Age ≥65 y, n (%)	50(47%)	572(58%)	.04
Male gender, n (%)	63(59%)	549(55%)	.5
Socioeconomic status, n (%)	…	…	
High	37(34%)	252(25%)	.03
Low	7(6.5%)	136(14%)	.03
CCI, median (IQR)	7 (4–10)	7 (4–10)	.7
CCI ≥ 3 points, n (%)	92(86%)	821(83%)	.4
Hypertension, n (%)	62(58%)	625(63%)	.3
Diabetes mellitus, n (%)	41(38%)	379(38%)	1
Ischemic heart disease, n (%)	26(24%)	297(30%)	.3
Chronic kidney disease, n (%)	37(34%)	384(39%)	.5
Hemodialysis, n (%)	9(8%)	110(11%)	.5
Hospital CDI diagnosis, n (%)	85 (79%)	665(67%)	.009
CDI toxin test, n (%)	…	…	
Immunoassay	66(62%)	522(53%)	.01
PCR	41(38%)	471(47%)	.01
Prior hospitalization within 90 d, n (%)	18(17%)	179(18%)	.9
Prior abdominal surgery, n (%)	5(5%)	22(2.2%)	.2
Previous antibiotic exposure within 90 d, n (%)	89 (83%)	693(70%)	.003
Concurrent non-CDI antibiotics, n (%)	59(55%)	462(46%)	.1
PPI treatment, n (%)	66(62%)	616(62%)	1
WBC count (K/µL), median (IQR)	6.4 (4.3–9.8)	9.5 (6.2–14.4)	<.001
WBC count > 15 K/µL, n (%)	9(8%)	181(18%)	.001
Creatinine level (mg/dL), median (IQR)	0.79 (0.61–1.27)	0.97 (0.7–1.7)	.001
Albumin level (mg/dL), median (IQR)	3 (2.5–3.3)	2.8 (2.4–3.3)	.3
Albumin level <3 mg/dL, n (%)	46(43%)	393(40%)	.2
**I**CH categories	…	…	
Hematologic malignancies and HSCT, n (%)	15 (14%)	202 (20%)	.02
HIV positive patients, n (%)	0 (0%)	7 (0.7%)	.02
SOT, n (%)	13 (12%)	81 (8%)	.02
Active solid tumor, n (%)	64 (60%)	464 (47%)	.02
Other conditions on IST, n (%)	15 (14%)	240 (24%)	.02

Abbreviations: IQR, interquartile range; CCI, Charlson comorbidity index; CDI, *C. difficle* infection; PCR, polymerase chain reaction; PPI, proton pump inhibitor; WBC, white blood cell. CRP, C reactive protein; ICH, immunocompromised; HSCT, hematopoietic stem cell transplant; SOT, solid organ transplantation; HIV, human immunodeficiency virus; IST, immune suppressive treatment.

**Figure 3.**
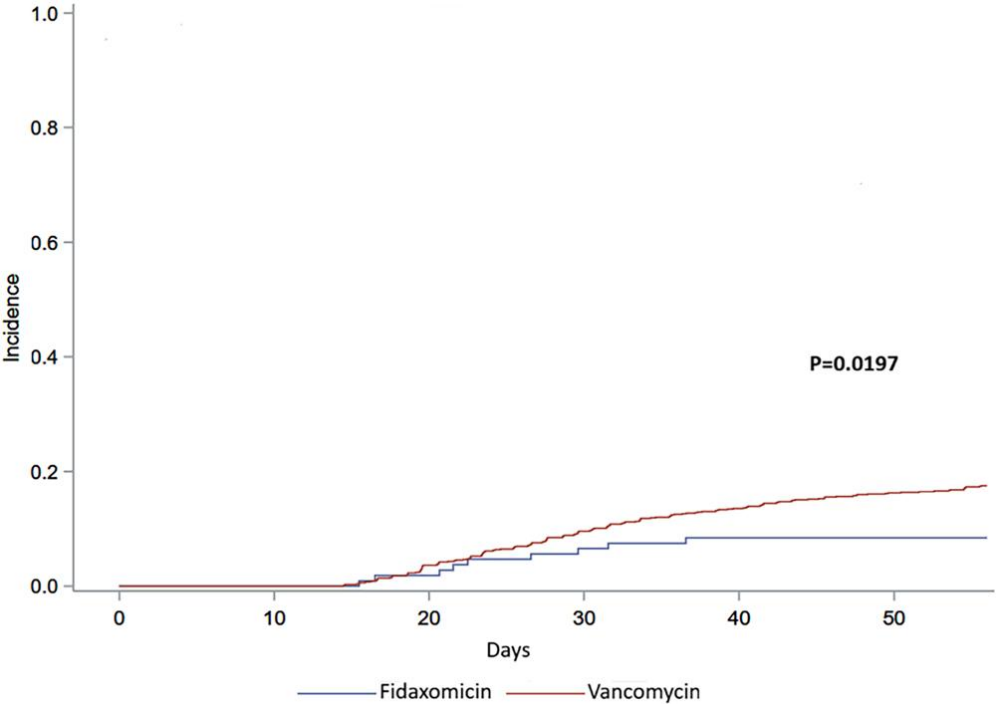
Incidence of CDI recurrence within 8 wks in ICHs treated with fidaxomicin versus vancomycin.

Table 4 presents the primary and secondary outcomes by treatment group. ICHs treated with fidaxomicin experienced fewer CDI-related complications, as defined by the composite outcome of CDI recurrence, colectomy, or 90-day mortality 24% (26/107) versus 34% (337/994), *P* = .051. Regarding secondary outcomes, the fidaxomicin group had a significantly lower recurrence rate within 8 weeks following diagnosis 8% (9/107) versus 17% (173/994), *P* = .019. There were no significant differences between the two groups in all-cause 90-day mortality 16% (17/107) versus 19% (187/994), *P* = .5 or post CDI colectomy rates 0.9% (1/107) versus 1.1% (11/994), *P* = 1. To minimize the potential influence of temporal factors, we conducted a focused analysis on the more recent period since 2018 (postfidaxomicin introduction). The analysis showed a significantly lower recurrence rate with fidaxomicin, HR = 0.49, 95% CI .25–.99, *P* = .047. In addition, we performed sensitivity analysis, with matching 1:1 and 1:2 (fidaxomicin: vancomycin) according to age, gender, Charlson comorbidity index, year of CDI antibiotic treatment, and ICH category. We found that fidaxomicin was significantly associated with lower recurrence rate as compared to vancomycin, with HRs of 0.41 (95% CI .18–.89, *P* = .025) and 0.44 (95% CI .21–.92, *P* = .029) respectively. Figure 3 displays the cumulative incidence of CDI recurrence within 8 weeks in ICHs treated with fidaxomicin versus vancomycin.

**Table 4.** Primary and Secondary Outcomes by Treatment group

Variable	Fidaxomicin Group (n = 107)	Vancomycin Group (n = 994)	*P* Value
Primary outcome (composite ^a^)	27 (25%)	371(37%)	.01
CDI recurrence	9(8%)	173(17%)	.02
90-d mortality	17(16%)	187(19%)	.5
Post cdi colectomy	1(0.9%)	11(1.1%)	1

^a^Composite outcome of complications includes CDI recurrence in 8 wks or 90-d mortality, or colectomy during 8 wks after 1st CDI episode.

CDI—*C. difficle* Infection.

### The Primary and Secondary Outcomes of *C. difficle* Infection in Immunocompromised Hosts

Univariable analysis of risk factors for the composite outcome of complications is presented in Table 4. Among ICHs with a first episode of CDI, 30% (687/2362) experienced complications, defined by the composite outcome of CDI recurrence, colectomy, or 90-day mortality. Patients in the complicated group were older (median age 71 vs 67 years, *P* < .01), had higher Charlson Comorbidity Index (CCI) (median CCI 8 vs 7, *P* < .01), and were more often diagnosed in the hospital setting 66% (455/687) versus 52% (870/1675), *P* < .01.

Patients without prior hospitalizations within 90 days or abdominal surgeries were less likely to develop CDI complications 25% (425/1675) versus 19% (128/687), *P* < .01; and 4% (61/1675) versus 1.3% (9/687), *P* < .01, respectively. The complicated group also had higher rates of previous antibiotic exposure 72% (498/687) versus 64% (1074/1675), *P* < .001, proton pump inhibitor (PPI) use 64% (440/687) versus 59% (986/1675), *P* = .02, and concurrent non-CDI antibiotic use at the time of diagnosis 45% (307/687) versus 34% (573/1675), *P* < .001.

Laboratory parameters at the time of CDI diagnosis also differed between groups. Patients in the complicated group had higher white blood cell counts (median 9.8 K vs 8.6 K, *P* < .01), were more likely to have hypoalbuminemia <3 mg/dL 46% (319/687) versus 24% (401/1675), *P* < .01 and had elevated CRP levels (median 10.2 vs 7.6 mg/dL, *P* = .03) (Table 5).

**Table 5.** Univariable Analysis of Risk Factors for the Composite Outcome of Complications in the Immunocompromised Host Group

Variable	Complicated Group (n = 687)	Non Complicated Group (n = 1675)	Univariate Analysis	*P* Value
Age, median (IQR)	71 (62–81)	67 (52–76)	1.01(1.02–1.03)	<.01
Age ≥65 y, n (%)	471 (69%)	907 (54%)	1.84 (1.52–2.22)	<.01
CCI, median (IQR)	8 (6–11)	7 (3–9)	1.1 (1.08–1.13)	<.01
CCI >3 points, n (%)	617 (90%)	1357 (81%)	2.04 (1.55–2.69)	<.01
Hospital CDI diagnosis, n (%)	455 (66%)	870 (52%)	1.9 (1.6–2.4)	<.01
Male gender, n (%)	381 (55%)	972 (58%)	0.9 (0.7–1.1)	.3
Low socioeconomic status, n (%)	95 (14%)	196 (12%)	1.3 (0.9–1.7)	.3
PCR positive *C. difficle* toxin, n (%)	282 (41%)	730 (44%)	0.9 (0.7–1.1)	.5
Hypertension, n (%)	476 (69%)	997 (60%)	1.5 (1.3–1.8)	<.01
Diabetes mellitus, n (%)	293 (43%)	633 (38%)	1.2 (1.02–1.5)	.03
Ischemic heart disease, n (%)	240 (35%)	457 (27%)	1.4 (1.2–1.7)	<.01
Chronic kidney disease, n (%)	285 (41%)	566 (34%)	1.4 (1.2–1.7)	<.01
Hemodialysis, n (%)	68 (10%)	160 (10%)	1.04 (0.8–1.4)	.8
Prior hospitalization within 90 d, n (%)	128 (19%)	425 (25%)	0.67 (0.54–0.84)	<.01
Prior abdominal surgery, n (%)	9 (1.3%)	61(4%)	0.36 (0.18–0.73)	<.01
Previous antibiotic exposure within 90 d, n (%)	498 (72%)	1074 (64%)	1.47 (1.21–1.78)	<.001
Concurrent non-CDI antibiotics, n (%)	307 (45%)	573 (34%)	1.55(1.29–1.86)	<.001
PPI treatment, n (%)	440 (64%)	986 (59%)	1.2 (1.03–1.5)	.02
WBC count (K/µL), median (IQR)	9.8 (6.1–14.8)	8.6 (5.6–12.6)	1.01 (1.003–1.02)	<.01
WBC count ≥15 K/µL, n (%)	129 (19%)	191(11%)	1.57 (1.22–2.03)	<.01
Creatinine level (mg/dL), median (IQR)	1 (0.71–1.71)	0.92 (0.7–1.47)	1.03 (0.96–1.1)	.4
Albumin level (mg/dL), median (IQR)	2.7 (2.3–3.1)	3.1 (2.6–5.1)	0.41 (0.34–0.49)	<.01
Albumin level <3 mg/dL, n (%)	319 (46%)	401 (24%)	0.34 (0.27–0.43)	<.01
CRP (mg/dL), median (IQR)	10.23 (4.1–20.3)	7.6 (2.7–16.8)	1.003 (1–1.01)	.03
Fidaxomicin treatment, n (%)	26 (4%)	81 (5%)	0.63 (0.4–1.003)	.051

Abbreviations: IQR, interquartile range; CCI, Charlson comorbidity index; CDI, *C. difficile* infection; PCR, polymerase chain reaction; PPI, proton pump inhibitor; WBC, white blood cell. CRP, C reactive protein.

In the multivariable analysis, treatment with fidaxomicin demonstrated a trend toward reducing the composite outcome of complications, with an adjusted odds ratio (aOR) of 0.63 (95% CI, .38–1.1; *P* = .07), although this did not reach statistical significance. Age over 65, previous antibiotic exposure within 90 days, and hypoalbuminemia (serum albumin <3 g/dL) were all significantly associated with the composite outcome. The corresponding adjusted odds ratios were 1.73 (95% CI, 1.22–2.46) for age, 1.48 (95% CI, 1.03–2.13) for previous antibiotic use, and 2.27 (95% CI, 1.64–3.13) for hypoalbuminemia, as shown in Table 6.

**Table 6.** Multivariable Analysis of Risk Factors for the Composite Outcome of Complications After 1st CDI Episode in Immunocompromised Patients

Variable	aOR	*P* Value
Fidaxomicin versus vancomycin	0.65 (0.39–1.07)	.09
Age ≥ 65 y versus <65	1.73 (1.22–2.46)	.002
CCI ≥ 3 versus <3 points	1.01 (0.59–1.71)	.9
Prior hospitalization within 90 d yes versus no	1.27 (0.74–2.15)	.4
Prior abdominal surgery yes versus no	0.63 (0.24–1.66)	.4
Albumin level < 3 versus ≥ 3 mg/dL	2.27 (1.64–3.13)	<.001
Previous exposure to antibiotics Yes versus no	1.48 (1.03–2.13)	.03

CCI, Charlson comorbidity index.

**Figure 4.**
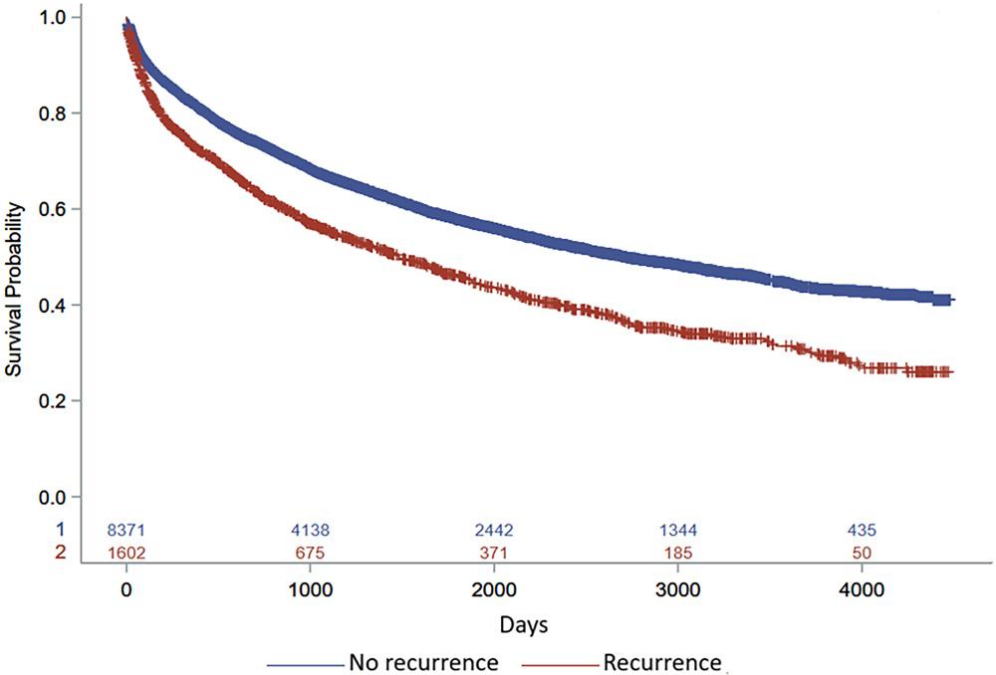
Long-term mortality among ICHs with CDI recurrence versus no recurrence.

In a separate multivariable analysis evaluating risk factors for CDI recurrence alone, fidaxomicin was significantly associated with a reduced risk of recurrent CDI within 8 weeks of the initial diagnosis, with an adjusted odds ratio of 0.44 (95% CI, .21–.96; *P* = .03). However, there was no significant association between fidaxomicin treatment and all-cause 90-day mortality, with an adjusted odds ratio of 0.86 (95% CI, .53–1.42; *P* = .56), as detailed in Table 7 and Supplementary Table 4. An interesting observation from the study was that long-term mortality was higher among patients who experienced CDI recurrence within 8 weeks compared to those who did not (*P* < .01), as illustrated in Figure 4.

**Table 7.** Multivariable Analysis of Risk Factors for CDI Recurrence After 1st CDI Episode in Immunocompromised Patients

Variable	Adjusted Hazard Ratio	*P* Value
Fidaxomicin versus Vancomycin	0.45 (0.21–0.95)	.04
Age ≥ 65 versus <65 y	1.47 (0.96–2.23)	.07
CCI ≥ 3 versus <3 points	0.61 (0.35–1.05)	.07
Prior hospitalization within 90 d yes versus no	1.37 (0.78–2.41)	.3
Prior abdominal surgery yes versus no	1.03 (0.38–2.74)	.9
Albumin level < 3 versus ≥ mg/dL	1.09 (0.76–1.58)	.6
Previous exposure to antibiotics yes versus no	1.75 (1.11–2.76)	.02

CCI, Charlson comorbidity index.

## DISCUSSION

While fidaxomicin was prescribed to only a small fraction (5%) of ICHs, its use was associated with a significantly reduced CDI recurrence rate (8% vs 17%, *P* = .019) and a trend toward fewer complications (24% vs 34%, *P* = .051).

These findings align with prior studies that have suggested a recurrence benefit with fidaxomicin in ICHs, particularly in a post hoc analysis of cancer patients [9] and in a recent retrospective study of ICHs [10]. However, as noted in the Introduction, not all studies have demonstrated this benefit, with some research on hematopoietic stem cell transplant (HSCT) and solid organ transplant (SOT) recipients showing no significant difference in recurrence rates [11]. In addition, a retrospective study from France including 397 high-risk patients where ICHs comprised 64% of the cohort (256/397), did not show a significant difference in recurrence rates between the fidaxomicin (10%) and vancomycin (9.2%) groups (*P* = .86) [13].

In addition to treatment choice, several other factors were independently associated with CDI-related complications in ICHs. Older age, previous antibiotic exposure within 90 days, and hypoalbuminemia (serum albumin <3 g/dL) emerged as significant predictors, with hypoalbuminemia demonstrating the strongest association (aOR 2.27). This finding underscores the importance of nutritional and inflammatory status as prognostic indicators in CDI outcomes. Moreover, patients who experienced CDI recurrence had significantly higher long-term mortality. A similar finding was reported by Tiseo *et al.* [8] in a multicenter retrospective study of 191 SOT recipients with first episode CDI, where the 8 weeks mortality rate without recurrence after the first CDI episode was 8.9%, but mortality increased in patients who developed a recurrent CDI (30.4%). This might be explained by the fact that CDI recurrence in ICHs might be representing the tip of the iceberg of the underlying debilitative health status, depth of immune suppression, and the presence of multiple comorbidities. However, attention should be focused on preventive strategies in this patient population to avoid recurrent CDI and associated poor outcomes.

### Top of Form

Notably, ICHs in our cohort were younger than immunocompetent patients but had significantly higher CCI scores, indicating a greater underlying disease burden. They were also more likely to be diagnosed during hospitalization and exhibited a higher prevalence of known CDI risk factors, including recent antibiotic use and PPI exposure. This emphasizes the distinct risk profile of ICHs, where immunosuppression, frequent healthcare interactions, and medication exposures intersect to elevate CDI risk.

Baseline laboratory findings were largely comparable between groups, although ICHs had lower white blood cell counts at diagnosis. This observation reinforces the need for clinicians to interpret laboratory values cautiously in ICHs, as conventional markers of infection severity may not accurately reflect disease burden in this population.

Taken together, our findings highlight the multifactorial nature of CDI outcomes in ICHs. Although recurrence rates were not universally higher in this group, specific subpopulations, particularly those with hypoalbuminemia or multiple risk factors, may benefit from tailored management strategies. The apparent benefit of fidaxomicin in reducing recurrence in ICHs adds important real-world evidence to the evolving discussion on optimal treatment strategies in this population. Given the cost and access barriers associated with fidaxomicin, future work should focus on defining which subgroups of ICHs derive the most benefit, thereby guiding more cost-effective and individualized treatment decisions. Prospective studies in diverse ICH populations will be essential to inform guideline development and improve outcomes in this growing and vulnerable group.

## Supplementary Material

ofaf751_Supplementary_Data

## Supplementary Data

Supplementary materials are available at *Open Forum Infectious Diseases* online. Consisting of data provided by the authors to benefit the reader, the posted materials are not copyedited and are the sole responsibility of the authors, so questions or comments should be addressed to the corresponding author.
