# Supplementary material for: Fidaxomicin for Initial Episode of Clostridioides difficile Infection Reduces Recurrence in Immunocompromised Hosts—a Large Retrospective Cohort Study
Source: Open Forum Infect Dis. 2025 Dec 11;12(12):ofaf751. doi: 10.1093/ofid/ofaf751 (PMC12719386; doi:10.1093/ofid/ofaf751)
Supplement: ofaf751_Supplementary_Data [file ofaf751_supplementary_data.docx]

Appendix

Tables

*Supplementary table1.* Immunocompromising conditions definitions*....................................* 2

*Supplementary table2.* Immunosuppressive drugs categories .................................*...............* 3

*Supplementary table3.* Baseline characteristics, CDI clinical course and outcomes according to the immunosuppression categories.........................................................................................*...................*4

*Supplementary table4.* Supplementary Table 4. Multivariate analysis of risk factors for 90 days mortality after 1^st^ CDI episode in immunocompromised hosts…………………………………..*...*5

Figures

*Supplementary figure 1.* Immunosuppressive drug types and distribution*..............................*6

*Supplementary figure 2.* SOT recipients according to organ types*...........................................*7

Supplementary table1. Immunocompromising conditions definitions

| **Solid tumor** | **Active solid tumors on antineoplastic drugs (see table2)**  Tumors of skin, brain, head and neck, lungs, GI, prostate, gynecological, sarcomas, unknown primary |
| --- | --- |
| **Hematologic malignancy** | Acute Leukemias, active lymphomas on therapy, MM, CLL, CML |
| **SOT** | Heart, lung, liver, pancreas, kidney transplant recipients |
| **HSCT** | Autologous and allogeneic HSCT |
| **HIV+/AIDS** |  |
| **Other IC conditions** | Other autoimmune and inflammatory diseases that are treated with one or more of immunosuppressive drug (table 2) |

GI – gastrointestinal, MM – multiple myeloma , CLL – chronic lymphocytic leukemia, CML- chronic myeloid leukemia , SOT- solid organ transplantation , HSCT- hematopoietic stem cell transplantation- , IC- immunocompromised.

Supplementary table 2. Immunosuppressive drugs categories

| **Antineoplastic drugs:** | Bendamustine, Bleomycin, Cladribine, Busulfan, Carboplatin, Cisplatin, Cytarabine, Cyclophosphamide, Daunorubicin, Doxorubicin, Etoposide, Fludarabine, Idarubicin, Melphalan, Methotrexate, Mitoxantrone, Pentostatin, Vincristine |
| --- | --- |
| **Immunosuppressive/immunomodulator drugs:** | Azathioprine, Cyclophosphamide, Cyclosporine, Mercaptopurine, Methotrexate, Mycophenolate mofetil (MMF), Sirolimus, Tacrolimus |
| **Corticosteroids** | Prednisone, dexamethasone, hydrocortisone |
| **Antibodies:** | Adalimumab, Alemtuzumab, Anti-Thymocyte Globulin (ATG), Apolizumab, Basiliximab, Belimumab, Bevacizumab, Brentuximab, Brodalumab, Certolizumab, Cetuximab, Daclizumab, Eculizumab, Galiximab, Gemtuzumab-Ozagamicin, Golimumab, Ibritumomab-Tiuxetan, Infliximab, Ipilimumab, Lumiliximab, Muromonab, Natalizumab, Nivolumab, Ofatumumab, Panitumumab, Pembrolizumab, Pertuzumab, Ramucirumab, Rituximab, Tocilizumab, Tositumomab, Trastuzumab, Ustekinumab, Vedolizumab, Zanolimumab |
| **Small molecules:** | Abatacept, Anakinra, Bortezomib, Bosutinib, Dasatinib Erlotinib, Etanercept, Everolimus ,Ibrutinib, Idelalisib, Imatinib, Ivosidenib, Lapatinib, Leflunomid, Lenalidomide, Midostaurin, Nilotinib, Pazopanib, Pomalidomide Regorafenib, Ruxolitinib ,Sorafenib, Sunitinib, Thalidomide, Tretinoin, Vismodegib |

Supplementary Table 3. Baseline characteristics, CDI clinical course and outcomes according to the immunosuppression categories

| **Variable** | **Immuno-competent**  **(n=8,842)** | **Active solid tumor**  **(n=1,109)** | **HM and HSCT**  **(n=469)** | **SOT**  **(n=175)** | **Patients on IST**  **(n=590)** | **HIV**  **(n=19)** | **P**  **value** |
| --- | --- | --- | --- | --- | --- | --- | --- |
| Age, median (IQR) | 74 (56-84) | 70 (62-78) | 74(63-83) | 61(46-68) | 59(33-74) | 50 (35-77) | <0.001 |
| Age > 65 yrs, n (%) | 5758 (65%) | 735 (66%) | 333 (71%) | 62(35%) | 241(41%) | 7(37%) | <0.001 |
| CCI, median (IQR) | 6 (2-9) | 8 (5-10) | 8 (6-11) | 7(5-9) | 3 (1-7) | 8 (7-11) | <0.001 |
| CCI > 3 points, n (%) | 6601 (75%) | 1023(92%) | 444 (95%) | 160(91%) | 328(56%) | 19(100%) | <0.001 |
| Male gender, n (%) | 5551 (63%) | 649(59%) | 268 (57%) | 76(43%) | 351(59%) | 9(47%) | <0.001 |
| **Referring facility** |  |  |  |  |  |  |  |
| Hospital, n (%) | 3248 (37%) | 714(64%) | 254(54%) | 134(77%) | 213(36%) | 10(53%) | <0.001 |
| Community, n (%) | 4879 (55%) | 364(33%) | 186(40%) | 38(22%) | 367(62%) | 8(42%) | <0.001 |
| Nursing home, n (%) | 715 (8%) | 31(3%) | 29(6%) | 3(2%) | 10(2%) | 1(5%) | <0.001 |
| **Ethnicity** |  |  |  |  |  |  |  |
| Jews, n (%) | 8139 (92%) | 1028(93%) | 427(91%) | 141(81%) | 526(89%) | 19(100%) | <0.001 |
| Arab, n (%) | 703 (8%) | 81(7%) | 42(9%) | 34(19%) | 64(11%) | 0(0%) | <0.001 |
| **Socioeconomic** |  |  |  |  |  |  |  |
| High, n (%) | 1928 (22%) | 290(26%) | 113(24%) | 33(19%) | 157(27%) | 0(0%) | 0.002 |
| Low, n (%) | 1201 (14%) | 135(12%) | 69(15%) | 27(15%) | 58(10%) | 2(11%) | 0.002 |
| **CDI test method** |  |  |  |  |  |  |  |
| ELISA Toxin, n (%) | 6157 (70%) | 615(55%) | 296(63%) | 88(50%) | 338(57%) | 10(53%) | <0.001 |
| Toxin A/B PCR, n (%) | 2681 (30%) | 492(44%) | 173(37%) | 87(50%) | 251(43%) | 9(47%) | <0.001 |
| Nap1, n (%) | 4 (5%) | 2(0.18%) | 0(0%) | 0(0%) | 1(0.2%) | 0(0%) | <0.001 |
| Hypertension, n (%) | 5766 (65%) | 716(65%) | 337(72%) | 144(82%) | 266(45%) | 10(53%) | <0.001 |
| Diabetes mellitus, n (%) | 3403 (38%) | 438(39%) | 224(48%) | 96(55%) | 161(27%) | 7(37%) | <0.001 |
| Ischemic heart disease, n (%) | 2850 (32%) | 319(29%) | 172(37%) | 78(45%) | 124(21%) | 4(21%) | <0.001 |
| Chronic kidney disease, n (%) | 2681 (30%) | 313(28%) | 207(44%) | 153(87%) | 172(29%) | 6(32%) | <0.001 |
| Hemodialysis, n (%) | 431 (5%) | 48(4%) | 27(6%) | 114(65%) | 38(6%) | 1(5%) | <0.001 |
| Pre-hospitalization, n (%) | 1882 (21%) | 264(24%) | 117(25%) | 30(17%) | 141(24%) | 1(5%) | 0.019 |
| Prior abdominal surgery, n (%) | 116 (1.3%) | 42(4%) | 6(1.3%) | 2(1.1%) | 20(3.3%) | 0(0%) | <0.001 |
| Prior antibiotics, n (%) | 5302 (60%) | 754(68%) | 311(66%) | 132(75%) | 363(59%) | 12(%) | <0.001 |
| Concurrent antibiotics, n (%) | 2435 (28%) | 458(41%) | 172(37%) | 84(48%) | 160(27%) | 6(32%) | <0.001 |
| Prior PPI in 6 months, n (%) | 3920 (44%) | 673(61%) | 281(60%) | 130(74%) | 334(57%) | 8(42%) | <0.001 |
| Prior statin in 6 months, n (%) | 5626 (64%) | 739(67%) | 328(70%) | 127(73%) | 286(48%) | 8(42%) | <0.001 |
| WBC count (K/µL), median (IQR) | 10.4(7.6-15.2) | 8.5 (4.88-13.18) | 9.5 (6.47-13.8) | 9.23 (5.75-14.56) | 9.2 (6.9-12.5) | 8.13(5.16-15.58) | <0.001 |
| WBC >15K/µL, n (%) | 1261 (14.3%) | 160(14%) | 70(15%) | 34(19%) | 52(9%) | 4(21%) | <0.001 |
| Creatinine mg/dl, mean+SD | 1.48+1.65 | 0.88 (0.67-1.36) | 1.03 (0.73-1.74) | 1.8 (1.14-3.35) | 0.85 (0.68-1.25) | 0.94 (0.7-1.72) | <0.001 |
| Albumin mg/dl, median (IQR) | 2.9 (2.5-3.5) | 2.83(2.4-3.3) | 2.9 (2.5-3.4) | 3 (2.47-3.5) | 3.26(2.70-3.87) | 3.1(2-4) | <0.001 |
| Albumin <3mg/dl, n (%) | 2011 (23%) | 405(37%) | 138(29%) | 61(35%) | 110 (19%) | 6(32%) | <0.001 |
| CRP mg/dl, median (IQR) | 9 (2.3-19) | 10.01(4.44-19.12) | 10.12 (4.27-19.35 | 8.6(2.88-15.81) | 4.24 (1.07-14.81) | 7.28 (1.2-18.51) | <0.001 |
| Fidaxomicin, n (%) | 47 (0.5%) | 64(6%) | 15(3%) | 13(7%) | 15(3%) | 0(0%) | <0.001 |
| Vancomycin, n (%) | 2820 (32%) | 464(42%) | 202(43%) | 81(46%) | 240(41%) | 7(37%) | <0.001 |
| Metronidazole, n (%) | 2702 (31%) | 325(29%) | 129(28%) | 44(25%) | 172(29%) | 7(37%) | <0.001 |
| Recurrent CDI within 8wks, n (%) | 1423 (16%) | 176(16%) | 70(15%) | 20(11%) | 79(13%) | 2(11%) |  |
| Post CDI colectomy, n (%) | 49 (0.5%) | 7(0.6%) | 5(1.1%) | 2(1.1%) | 6(1%) | 0(0%) | 0.471 |
| 30-day mortality, n (%) | 485 (5%) | 58(5%) | 40(9%) | 8(5%) | 20(3.4%) | 0(0%) |  |
| 90-day mortality, n (%) | 1322 (15%) | 217(20%) | 98(21%) | 19(11%) | 44(7%) | 1(5%) |  |

HM – hemato-oncology, HSCT- hematopoietic stem cell transplant , SOT -Solid organ transplantation, IST - Immunosuppressive therapy, HIV – human immunodeficiency virus, IQR -interquartile range , CCI -Charlson comorbidity index, ELISA – enzyme linked immunosorbent assay, PCR –polymerase chain reaction, NAP1 - nucleosome assembly protein 1, PPI – proton pump inhibitor, WBC – white blood cell, SD – standard deviation, CRP- C-reactive protein, CDI - *Clostridioides difficle* infection.

Supplementary Table 4. Multivariate analysis of risk factors for 90 days mortality after 1^st^ CDI episode in immunocompromised hosts

| Variable | aOR | P value |
| --- | --- | --- |
| Fidaxomicin vs vancomycin | 0.89 (0.53-1.47) | 0.65 |
| Age > 65 years vs <65 years | 1.65 (1.14-2.39) | 0.007 |
| CCI > 3 points vs <3 | 2.22 (0.99-4.97) | 0.051 |
| Prior hospitalization yes vs no | 1.16(0.64-2.09) | 0.6 |
| Prior abdominal surgery yes vs no | 0.57 (0.18-1.81) | 0.35 |
| Albumin level < 3mg/dl vs >3mg/dl | 2.93 (2.001-3.28) | <0.01 |
| Prior antibiotics | 1.20 (0.82-1.75) | 0.3 |

CCI- Charlson Comorbidity Index, aOR – adjusted odds ratio.

Supplementary Figure 1. Immunosuppressive drug types and distribution (see drug details in S.table 2).

Supplementary figure 2. SOT recipients according to organ types (n=175)
